# *In-vivo* and numerical analysis of the eigenmodes produced by a multi-level Tic-Tac-Toe head transmit array for 7 Tesla MRI

**DOI:** 10.1371/journal.pone.0206127

**Published:** 2018-11-27

**Authors:** Tales Santini, Yujuan Zhao, Sossena Wood, Narayanan Krishnamurthy, Junghwan Kim, Nadim Farhat, Salem Alkhateeb, Tiago Martins, Minseok Koo, Tiejun Zhao, Howard J. Aizenstein, Tamer S. Ibrahim

**Affiliations:** 1 Department of Bioengineering, University of Pittsburgh, Pittsburgh, PA, United States of America; 2 Siemens Medical Solutions, Pittsburgh, PA, United States of America; 3 Department of Psychiatry, University of Pittsburgh Medical Center, Pittsburgh, PA, United States of America; Linköping University, SWEDEN

## Abstract

Radio-frequency (RF) field inhomogeneities and higher levels of specific absorption rate (SAR) still present great challenges in ultrahigh-field (UHF) MRI. In this study, an in-depth analysis of the eigenmodes of a 20-channel transmit Tic-Tac-Toe (TTT) RF array for 7T neuro MRI is presented. The eigenmodes were calculated for five different Z levels (along the static magnetic field direction) of the coil. Four eigenmodes were obtained for each Z level (composed of 4 excitation ports), and they were named based on the characteristics of their field distributions: quadrature, opposite-phase, anti-quadrature, and zero-phase. Corresponding finite-difference time-domain (FDTD) simulations were performed and experimental B_1_^+^ field maps were acquired using a homogeneous spherical phantom and human head (in-vivo). The quadrature mode is the most efficient and it excites the central brain regions; the opposite-phase mode excites the brain peripheral regions; anti-quadrature mode excites the head periphery; and the zero-phase mode excites cerebellum and temporal lobes. Using this RF array, up to five eigenmodes (from five different Z levels) can be simultaneously excited. The superposition of these modes has the potential to produce homogeneous excitation with full brain coverage and low levels of SAR at 7T MRI.

## Introduction

Ultrahigh-field (UHF) magnetic resonance imaging (MRI) can be exploited for medical research and applications through its higher resolution anatomical imaging, inherent higher contrast, and improved spectroscopy. However, there are technical and physical challenges associated with UHF imaging that have not been completely addressed yet: a) the inhomogeneous distribution of the circularly polarized transmit fields (B_1_^+^), responsible for excitation [1–4]; b) the potentially higher power deposition in the tissues [5, 6]; c) the absence of commercial transmit body coil integrated into the system (commonly seen at lower fields); and d) the difficulty to supervise the local specific absorption rate (SAR) [7].

Several designs of radio-frequency (RF) transmit arrays have been proposed to improve the RF (B_1_^+^ and SAR) performance at UHF MRI [8–11]. A major advantage of these multichannel systems is that the channels of the RF arrays can be manipulated to operate at specific amplitudes and phases, having the potential to optimize a certain characteristic of the RF fields distribution (usually improving B_1_^+^ homogeneity and/or efficiency and minimizing SAR.) To determine these operational points, some techniques have been applied, among them the eigenmodes approach; for instance, the two-dimension image uniformity of a spherical phantom was 10% by linearly combining four harmonics modes [12]. Moreover, two time-interleaved acquisitions using different modes have shown improvement in the homogeneity without increasing the time of acquisition [13]. Eigenmode approaches have also been utilized to analyze the signal-to-noise ratio (SNR) behavior of phased array receive coils [14, 15] and to increase the acceleration factor in parallel imaging [16].

In this work, a description and an excitation paradigm are presented for a 20-channel, five-sided Tic-Tac-Toe (TTT) RF transmit array design for 7 Tesla (T) MRI [9]. The RF coil performance (B_1_^+^ and SAR) was studied using the eigenmodes approach. The modes were numerically calculated from finite-difference time-domain (FDTD) simulations and experimentally verified in-vivo and on a spherical phantom with a 7T human MRI scanner. Using the designed RF array, up to five eigenmodes can be excited simultaneously. The combination of these eigenmodes has the potential to achieve an efficient and homogeneous B_1_^+^ field distribution with low levels of SAR at UHF MRI.

## Material and methods

### The RF array design and construction

**Fig 1:**
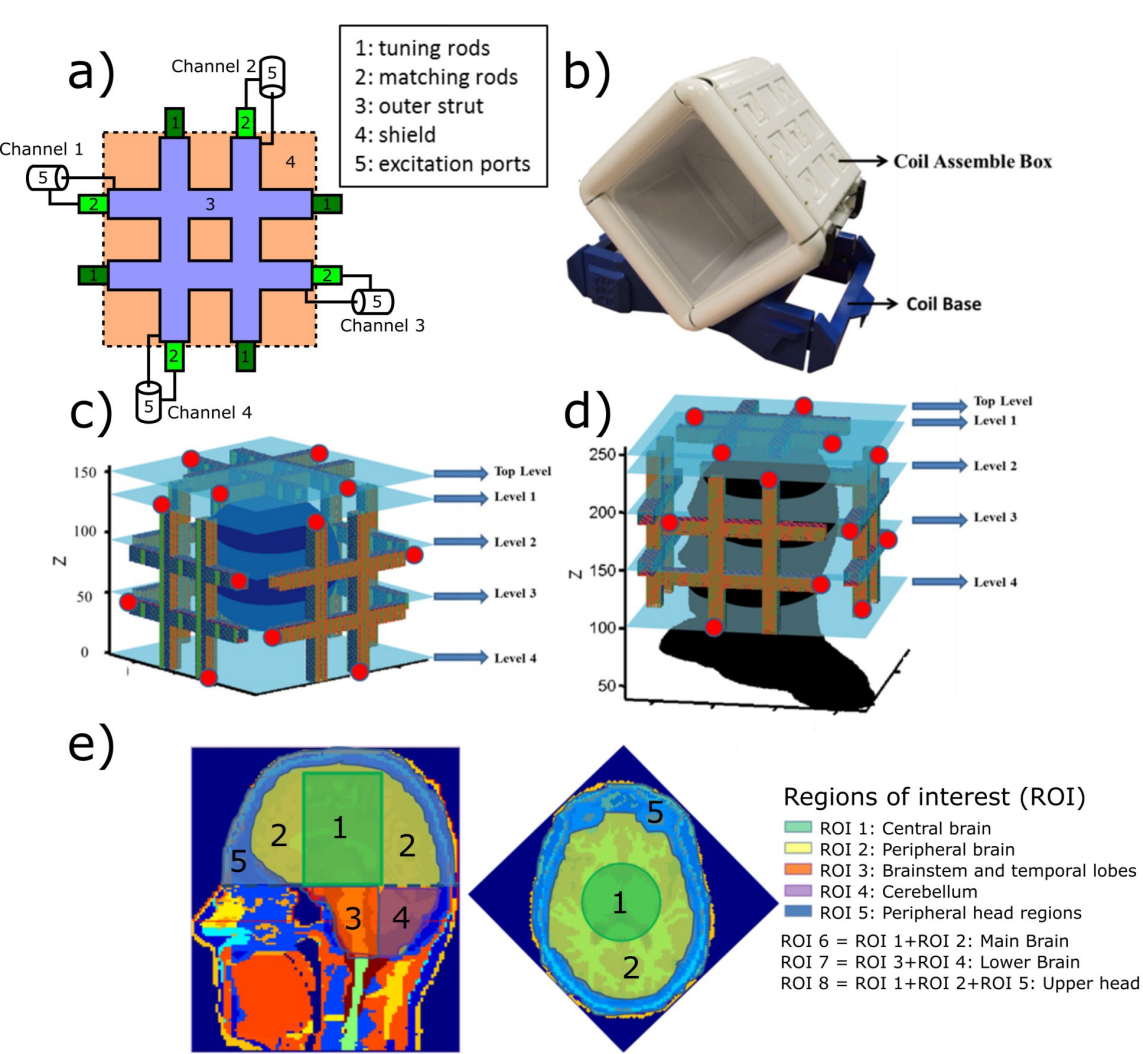
Coil schematic diagram, load position and regions of interest. In (a), the schematic diagram of a four-element, 2x2 Tic-Tac-Toe array design. The copper rods (1 and 2) are partially inside the copper struts (3) providing matching and tuning to the RF coil. In (b), the assembled RF coil system, composed of 5 sets/sides of the 2x2 Tic-Tac-Toe transmit arrays (total of 20 transmit elements.) In (c), FDTD spherical (~17cm in diameter) water phantom model (108 by 108 by 108 Yee cells with isotropic resolution of ~1.6mm). The red dots indicate the excitation points of the three visible sets of the 2x2 Tic-Tac-Toe arrays. The 5 levels of the coil in Z direction are shown. In (d), the Duke Virtual Family Adult Head Model (114 by 117 by 144 Yee cells with isotropic resolution of ~1.6mm). In (e), the head model was divided into 8 different regions of interest (ROI) as indicated by the color code and the numbers.

The TTT coil design has been applied to several UHF human MRI applications, including head [9, 17–19], breast [20, 21], torso [22], and foot [23, 24]. Fig 1(A) shows the schematic diagram of a four-element 2x2 TTT transmit array design. The coil is composed of eight square-shaped transmission lines electrically connected to each other in a tic-tac-toe fashion. The outer strut was built from 8*μ*m-thick single-sided copper sheets (Polyflon, Germany). The inner rods are composed of solid square-shaped copper (McMaster-Carr, USA) partially inserted into the outer strut, creating a squared shape coaxial transmission line. The dimensions of the outer strut are 228.6 × 228.6 × 19.0 mm^3^.

The excitation ports of one side (four channels) are also shown in Fig 1(A). Tuning and matching of the coil is performed by changing the length of the inner rods inside the outer struts, presenting similar performance in terms of s-parameters as the demonstrated in [24]. The RF copper shielding is located at the back of the coil struts (with a gap of 15.8mm) and it functions as the ground of a cavity resonator, being responsible for both increasing the RF efficiency and preventing RF leaking. The RF copper shielding is composed of double layer 4μm thick copper sheets (Polyflon, Germany) and it was slotted with specific patterns to reduce eddy currents while the RF performance is maintained, as demonstrated in [25]. The non-metal parts of the array were 3D printed using polycarbonate (Stratasys, USA).

Fig 1(B) shows the assembled RF coil system which is composed of five sides of the four-element 2x2 Tic-Tac-Toe transmit array (earlier described), resulting in a total of twenty transmit channels/excitation ports. The channels of the RF array were tuned and matched on the bench using the Agilent Network Analyzer Model E5062A (Santa Clara, US). While the five sides of the four-element 2x2 TTT transmit array are inherently decoupled from each other (less than -16dB), on any 2x2 side, the coupling among the adjacent transmit channels (S12 and S14) is about -9 to -11 dB, and the coupling between opposite elements (S13) is about -3 to -4 dB.

### FDTD simulations

An in-house FDTD software package with an accurate transmission-line feed mechanism [26] was implemented to model the RF performance of the 20-channel TTT transmit array. This simulation package has been previously utilized and verified [20, 21, 26–32]. The RF fields were calculated with the coil loaded with a homogeneous spherical phantom model (diameter = 17.1cm, conductivity = 0.46 S/m and relative permittivity = 79.0) and a human head model (18.2*cm* × 18.7*cm* × 23.0*cm*), which was extracted from the Virtual Family Duke Model [33]. The resolution of the models is ~1.6mm isotropic per voxel and the simulation was run until a steady state was achieved (100,000 time steps, time resolution of 3 ps). Fig 1C and 1D shows respectively the relative position of the homogenous spherical phantom and the human head model inside the RF array. The excitation points are identified by the red dots.

The B_1_^+^ field distribution was analyzed in eight regions of interest (ROI) described in Fig 1(E). The ROIs are based on human head anatomical characteristics as well as the electromagnetic characteristics of the coil.

### Calculations of the eigenmodes

The current distributions induced on the RF coil elements can be controlled by manipulating the amplitude and phase of the voltages feeding the excitation ports. A specific current distribution induced on the elements of the RF coil also determines an eigenmode [34]. Consequently, the B_1_^+^ field distribution and SAR can be manipulated as a result of the superposition of fields produced by the individual elements. In this work, the set of B_1_^+^ field distributions was arranged by:

$$C=\left(\begin{array}{ccc} B_{1(1)}^{+} & \cdots & B_{L(1)}^{+}\\ \vdots & \ddots & \vdots \\ B_{1(n)}^{+} & \cdots & B_{L(n)}^{+} \end{array}\right)$$ (1)

where ***C*** is the B_1_^+^ field matrix generated by an array with *L* transmit channels, *n* is the number of Yee cells inside the ROI. ***C*** * ***C*** gives the correlation among the channels of the array; therefore, the eigenmodes can be calculated by:

(C*C)v=λv (2)

where ***v*** is a unitary matrix of eigenvectors; $\lambda =\left(\begin{array}{ccc} \lambda_{1} & \ldots & 0\\ \vdots & \ddots & \vdots \\ 0 & \ldots & \lambda_{L} \end{array}\right)$ is a diagonal matrix of eigenvalues. With solutions for Eq 2, ***Cv*** is the spatially pseudo-independent fields or eigenmodes of the transmit coil; ***v*** gives the phase and amplitude of each coil channel; *λ*_*i*_ represents the field energy for eigenmode *i*.

The transmit array was grouped into five levels of four elements along the static magnetic field (Z) direction: Top_Level, Level_1, Level_2, Level_3, and Level_4 (see Fig 1C and 1D). The eigenmodes were calculated in each Z level of the transmit array by applying Eq 2 on the simulated B_1_^+^ field distributions; thus totaling four different excitation field patterns per level and 20 in total. Since the magnetic field distribution and SAR are two major concerns for 7T imaging, the attributes of the modes and coil Z levels were evaluated using three criteria:

1. average B_1_^+^ intensities inside each ROI for each Z level and mode, scaled for 1W input power per channel (totaling 4W for one Z level);
2. B_1_^+^ homogeneity calculated by the coefficient of variation (CV) inside each ROI for each Z level and mode;
3. average and peak SAR over the whole head volume (from the top of the neck) for each Z level and mode, scaled for 1W input power per channel (totaling 4 W for one Z level).

Please note that IEC/FDA limits the SAR in 3.2 W/kg for 10g of tissue inside the human head [35]. SAR levels were therefore evaluated in terms of average SAR over the whole head volume, peak SAR over any 10g of tissues, and safety excitation efficiency (SEE) [36], defined as average B_1_^+^ intensity over the combined volume of all eight ROIs divided by the average SAR over the whole head volume $\left[\mu T\sqrt{kg}/\sqrt{W}\right]$.

The eigenmodes were combined using an optimization of the 20-channel B_1_^+^ fields. The optimization aims at minimizing the CV of the B_1_^+^ field distribution within the ROI that encapsulates the whole head above and including the cerebellum and excluding the nasal cavities. The resultant field distribution was then scaled by 1W of total input power and the SEE was calculated based on the average B_1_^+^ field in the ROI divided by the square root of the average SAR for the whole head.

### MRI experiments

The FDTD calculated eigenmodes were experimentally verified using the constructed 20-channel transmit array. The MR experiments were conducted using a 7 Tesla MRI scanner (Siemens MAGNETOM, Germany). This study was approved by the University of Pittsburgh’s Institutional Review Board (IRB PRO17030036). One healthy volunteer was scanned after signing a written informed consent. The phantom MRI imaging experiment and the in-vivo study were conducted by acquiring relative B_1_^+^ maps using Turbo Flash MRI sequence; the outputs of this MRI sequence are: 1) the B_1_^+^ distribution for each transmit channel (scaled to the square root of the sum of the square of all connected transmitting channels); and 2) the relative phases. The sequence parameters used were: TE/TR = 2.34/160ms, resolution 3.2mm isotropic, flip angle 12 degrees. The scanner is equipped with 8 channels in the parallel transmit (pTx) mode with 1kW power amplifier per channel (8kW in total). These 8 transmit-channels were connected to the RF array in 2 Z Levels (each level has 4 channels) in each B_1_^+^ mapping experiment. Level_1 (most homogeneous level) was always connected in addition to another level (Fig 1D) per one B_1_^+^ mapping measurement. The 4 transmit-channels not connected to Level_1 were manually changed to another level until all the B_1_^+^ maps were acquired for all (5) Z levels. A transmit/receive (T/R) switch box was used to receive the signal from all 20 channels for any B_1_^+^ mapping acquisition. The transmit channels of the coil that were not used in a specific B_1_^+^ mapping acquisition were terminated with 50Ω loads through the T/R box.

## Results

### Calculation of the eigenmodes

By applying Eq 2 on the FDTD-simulated B_1_^+^ fields, the phases and amplitudes of the eigenmodes were obtained for each Z level of the transmit array; the results are presented in Table 1. Four modes were identified, and these modes presented uniformly distributed relative phase shifts and constant amplitudes among the 4 channels of each Z level: Mode_1 (named as quadrature) presents phase increments of ~90°; Mode_2 (opposite-phase) has increments of ~180°; Mode_3 (anti-quadrature) presents increments of ~270°; and Mode_4 (zero-phase) has increments of ~0° or ~360°. There were minor phase (<8°) and amplitude (<8%) deviations among the 5 different Z levels, which is impacted by the position of the load inside the RF coil. For practical purposes, the phases were kept as multiples of 90° and the amplitudes were considered to be the same for all channels.

**Table 1:** FDTD-calculated relative phases and amplitudes associated with the Eigenmodes of the array’s five Z levels. The coil was loaded with the homogeneous spherical phantom.

		Mode1 (Quadrature)	Mode2 (Opposite-phase)	Mode3 (Anti-quadrature)	Mode4 (Zero-phase)
**Top Level**	Phases	(0°, 90.7°, 179.4°, 268.6°)	(0°, 181.1°, 0.3°, 179.3°)	(0°, 270.1°, 180.3°, 90.2°)	(0°, -0.1°, -0.6°, 0.5°)
	Amplitudes	(0.50, 0.50, 0.50, 0.50)	(0.49, 0.50, 0.51, 0.50)	(0.50, 0.50, 0.50, 0.50)	(0.50, 0.50, 0.50, 0.50)
**Level1**	Phases	(0°, 87.7°, 173.5°, 265.5°)	(0°, 179.4°, -0.5°, 180.1°)	(0°, 267.1°, 178.8°, 89.7°)	(0°, -2.3°, -7.3°, -4.7°)
	Amplitudes	(0.49, 0.53, 0.51, 0.47)	(0.50, 0.50, 0.50, 0.50)	(0.50, 0.50, 0.50, 0.50)	(0.51, 0.47, 0.49, 0.53)
**Level2**	Phases	(0°, 91.2°, 178.8°, 266.9°)	(0°, 181.1°, 5.4°, 184.1°)	(0°, 270.1°, 182.1°, 92.0°)	(0°, 0.8°, -4.5°, -4.6°)
	Amplitudes	(0.47, 0.54, 0.53, 0.46)	(0.50, 0.49, 0.50, 0.51)	(0.51, 0.50, 0.49, 0.50)	(0.52, 0.47, 0.48, 0.52)
**Level3**	Phases	(0°, 91.6°, 180.0°, 268.3°)	(0°, 180.5°, 1.3°, 180.7°)	(0°, 270.1°, 180.0°, 89.9°)	(0°, 1.3°, -1.2°, -2.4°)
	Amplitudes	(0.48, 0.51, 0.52, 0.49)	(0.50, 0.49, 0.50, 0.51)	(0.50, 0.50, 0.50, 0.50)	(0.51, 0.49, 0.49, 0.51)
**Level4**	Phases	(0°, 89.8°, 179.5°, 89.7°)	(0°, 179.8°, -0.4°, 179.8°)	(0°, 269.8°, 179.5°, 89.7°)	(0°, -0.2°, -0.8°, -0.5°)
	Amplitude	(0.50, 0.50, 0.50, 0.50)	(0.50, 0.50, 0.50, 0.50)	(0.50, 0.50, 0.50, 0.50)	(0.50, 0.50, 0.50, 0.50)

### B_1_^+^ field and SAR comparisons of the eigenmodes

#### B_1_^+^ field intensities and homogeneity of the eigenmodes

**Fig 2:**
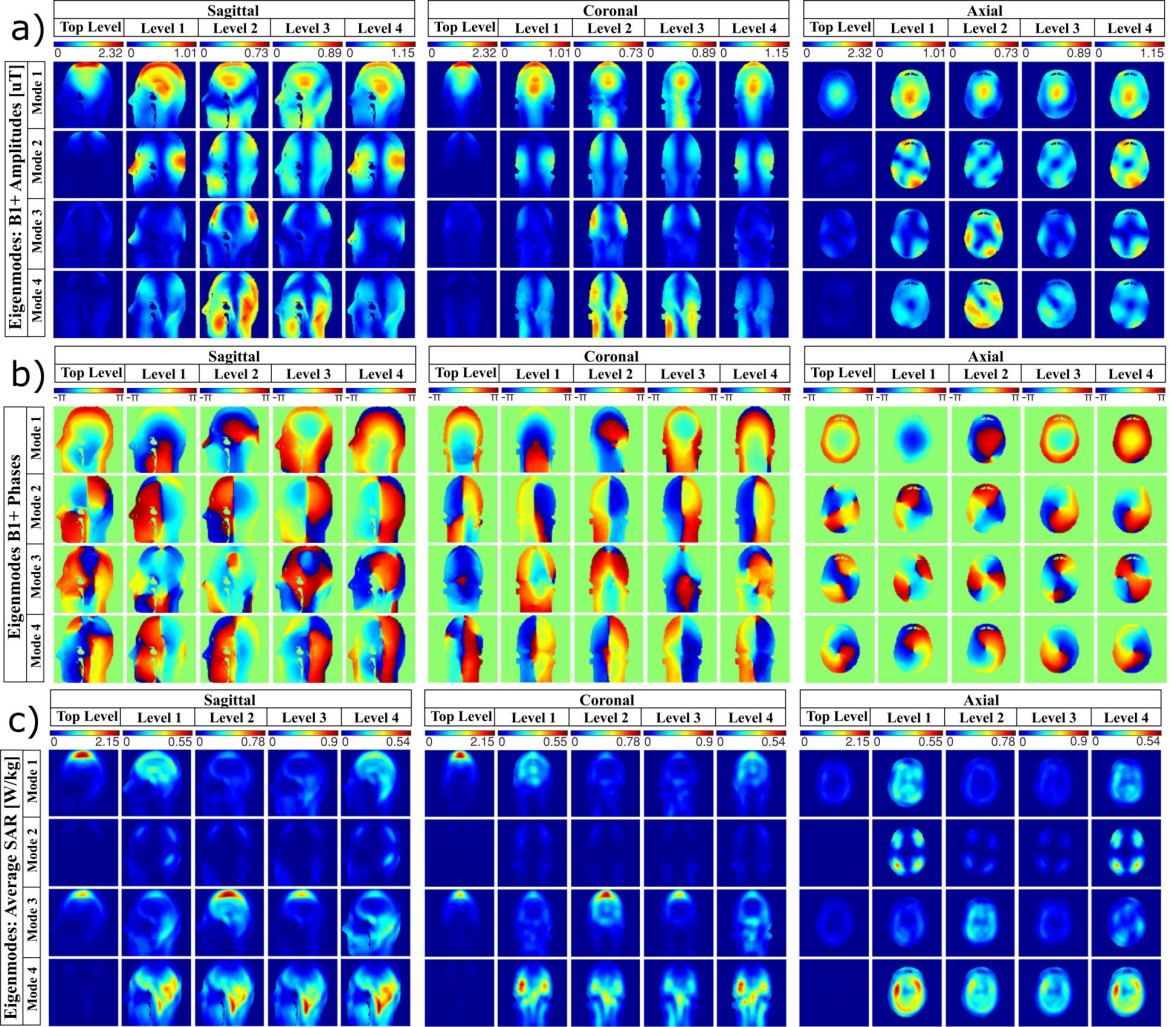
Simulated B_1_^+^ field and SAR distributions of the Eigenmodes in the Duke head model for each level (shown in Fig 1). The central slices in sagittal, coronal, and axial planes are shown. In (a), the amplitude of B_1_^+^ field distributions, in μT for 1W input power per channel (total 4W as each level contains 4 channels). For the four Eigenmodes per level, the colorbar is scaled from 0 to the maximum. In (b), the phase of the B_1_^+^ field distribution in radians. In (c), the SAR distributions in W/kg for 10g of tissues per 1W input power per channel (total 4W). The coil was loaded with the Duke Virtual Family Adult Head Model.

The FDTD-calculated B_1_^+^ field distribution of all modes for all the Z levels are presented in Fig 2(A). When comparing the eigenmodes in different Z Levels, the following observations are noted:

- Mode_1 (quadrature) generally provides high B_1_^+^ intensity in the central regions of the head/brain with the bright spot generally moving along the Z direction for distinctive Z levels;
- Mode_2 (opposite-phase) generates peripheral brain excitation;
- Mode_3 (anti-quadrature) generally excites the periphery of the head;
- Mode_4 (zero-phase) excites the lower brain (cerebellum and temporal lobes).

The B_1_^+^ field phase distribution maps are shown in Fig 2(B) (note that −2*π* = 2*π*, i.e., the intense blue color is equal to the intense red color in the colorbar).

**Fig 3:**
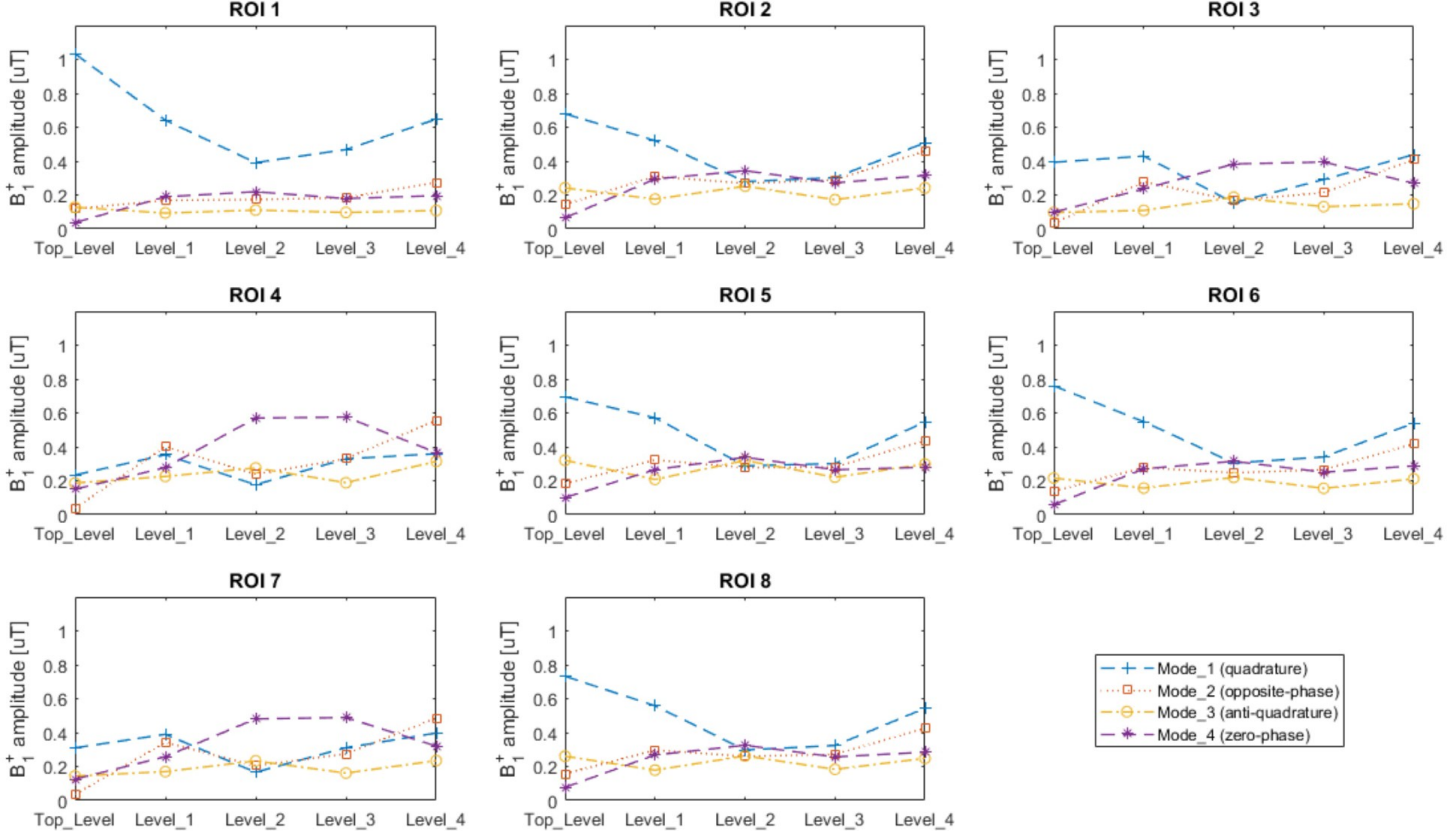
Average B_1_^+^ intensities calculated inside the 8 different regions of interest (ROIs) shown in Fig 1(E) for each Z level of the RF array shown in Fig 1(D). The scale is in μT for 1W input power per channel (total 4W).

The values of B_1_^+^ field intensities for all modes, levels, and ROIs are presented in Fig 3, from which we can note that:

- Top_level produces an efficient excitation in the upper head (ROIs 1, 2, 5, 6, and 8) when operating in Mode_1, presenting an average B_1_^+^ intensity of 0.73μT for 1W input power per channel (total 4 W) in these ROIs.;
- Level_1 and Level_4 are also efficient operating in Mode_1, producing an average B_1_^+^ of 0.54μT in the ROIs 1, 2, 3, 5, 6, and 8;
- Levels 2 and 3 produces an efficient excitation in the lower brain (ROIs 3, 4, and 7) when operating in Mode_4, presenting an average B_1_^+^ of 0.48μT in these regions.

**Fig 4:**
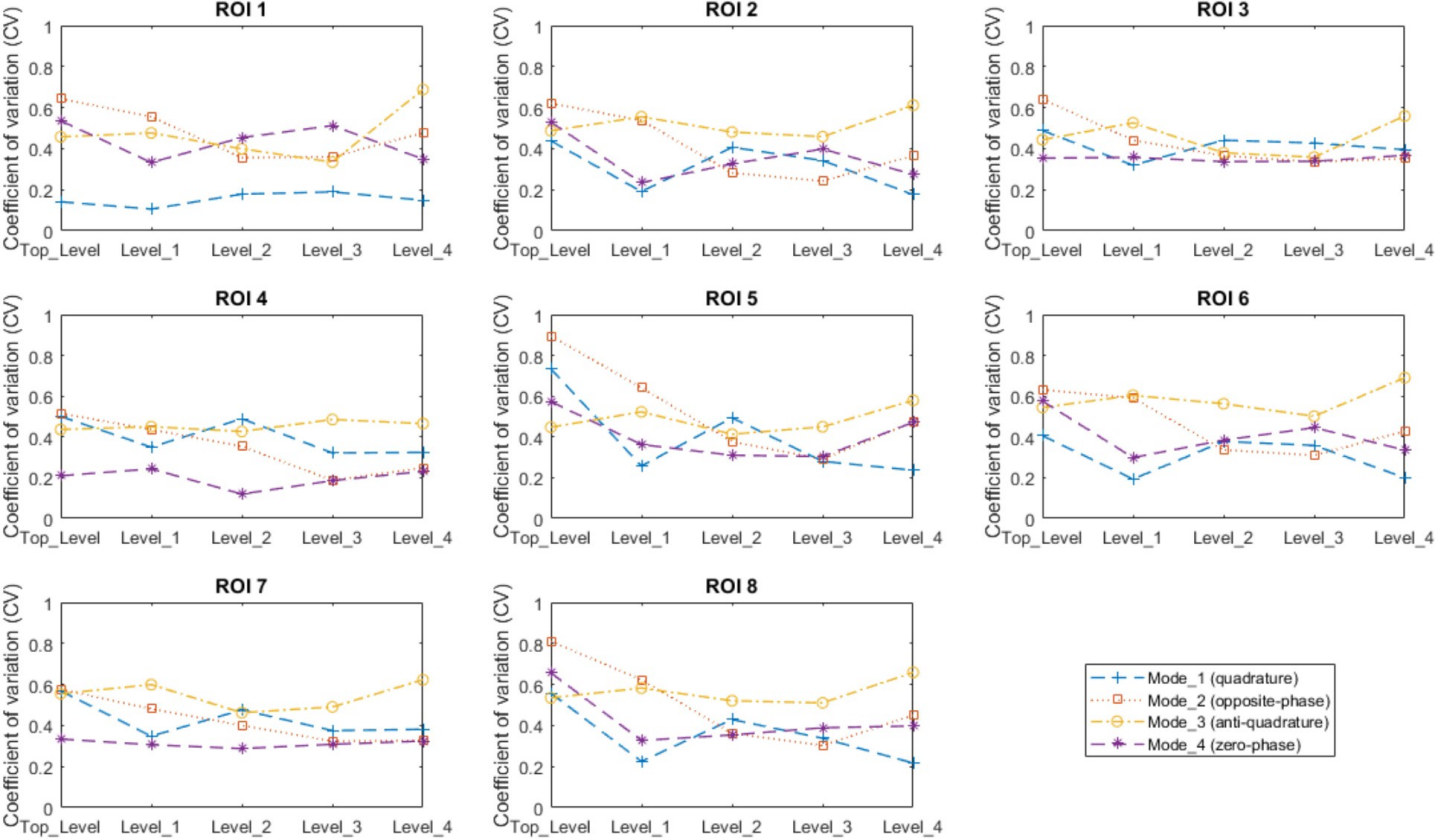
Coefficient of variation (standard deviation over the mean of B_1_^+^ field distribution) calculated inside the 8 different regions of interest (ROIs) shown in Fig 1(E) for each Z level of the RF array shown in Fig 1(D).

The CV of the B_1_^+^ field intensities over the specified eight ROIs for different modes and levels are shown in Fig 4.

#### SAR comparison for the eigenmodes at different Z levels

The numerically calculated SAR distributions for all eigenmodes from all Z levels are shown in Fig 2(C). Preferable modes present higher average B_1_^+^ intensity and lower peak and average SAR. The following observations are noted:

- the SAR distribution significantly varies for different eigenmodes and Z levels;
- the highest SAR regions usually correspond to lower intensities of B_1_^+^;
- Top_level operating in Mode_1 produces the highest peak SAR, but it is also B_1_^+^ efficient;
- Levels 1 and 4 produce homogeneous SAR distribution when operating in Mode_1;
- Mode_4 produces higher levels of SAR in the lower brain regions (except Top_level);
- Mode_1 usually produces low levels of average and peak SAR (except in Top_level) and high levels of SEE.

**Fig 5:**
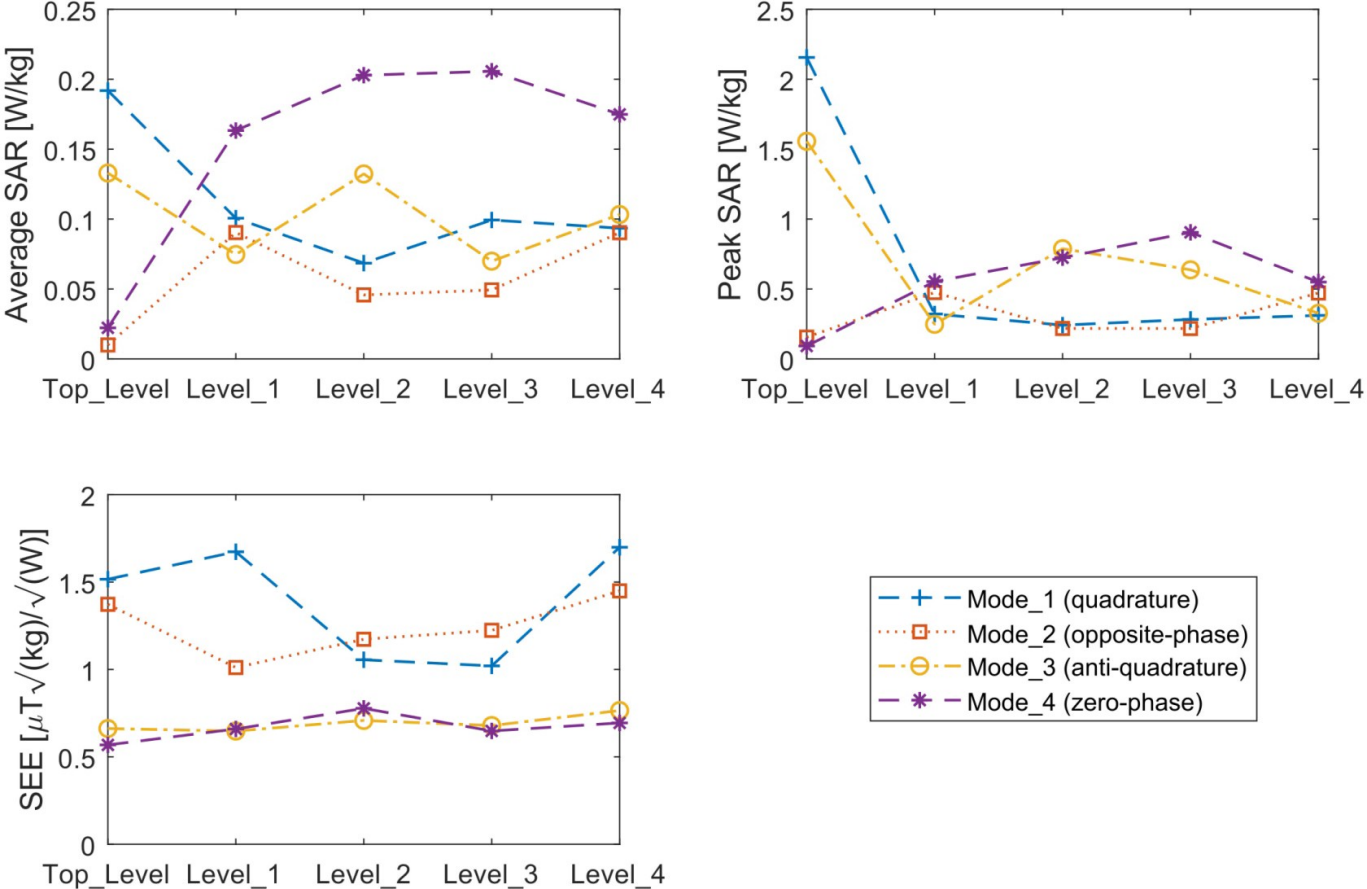
SAR evaluation of the Eigenmodes for each Z level of the RF array shown in Fig 1(D). In a) the average SAR per 1 W input power per channel (total 4 W). In b) the peak SAR per 1 W input power per channel (total 4 W). In c), the safety excitation efficiency (SEE) (the B_1_^+^ field is averaged over a volume that encapsulates all eight regions of interest.) The results are presented for the Duke Virtual Family Adult Head Model.

Fig 5 shows the average/peak SAR and SEE values for all Z levels and eigenmodes of the transmit array.

### Experimental verification

**Fig 6:**
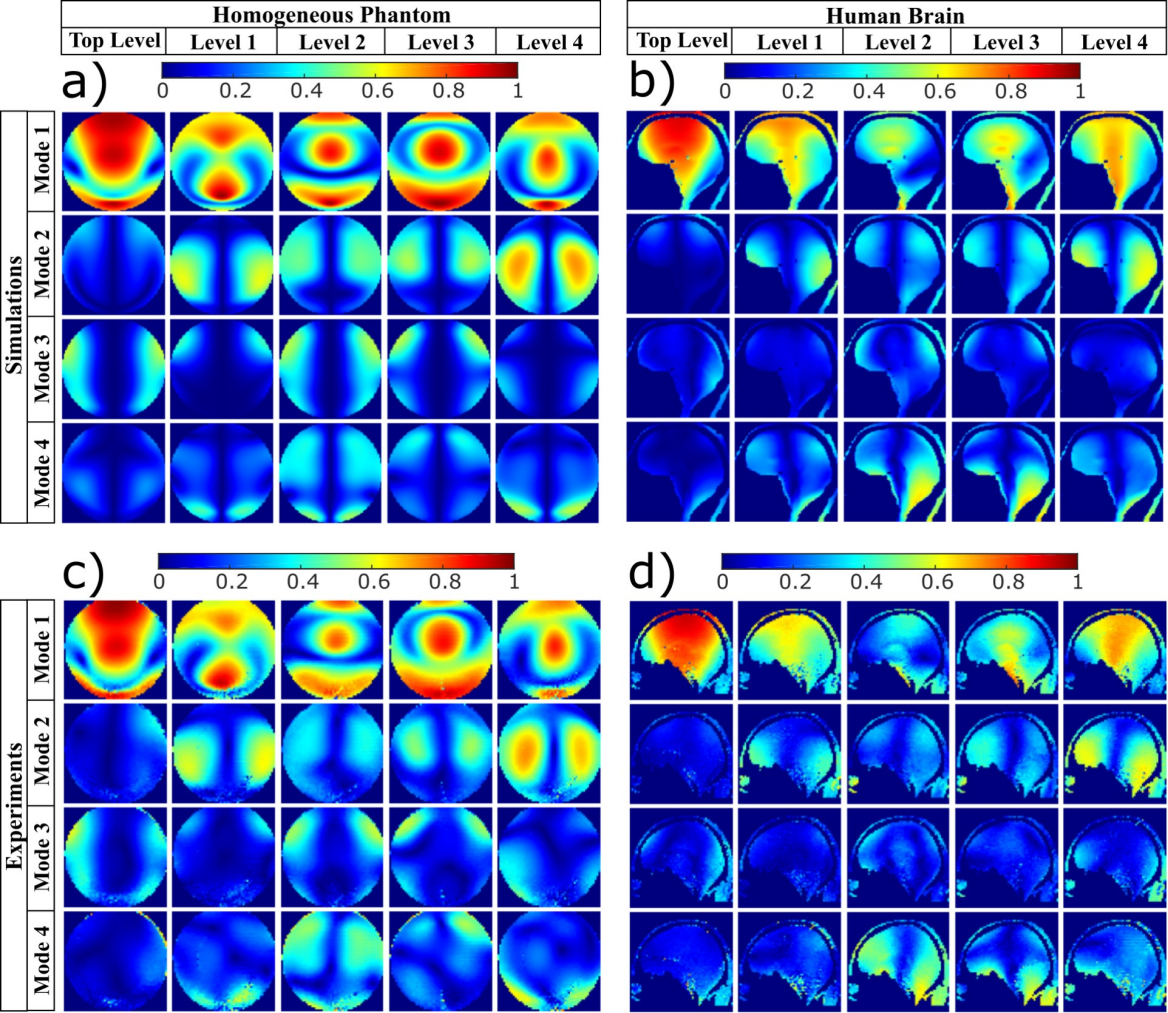
Experimental verification and simulated B_1_^+^ field distributions of the Eigenmodes for the homogenous spherical phantom and the human head, showing the central sagittal view. In (a), the simulated B_1_^+^ field distributions in the homogeneous spherical phantom with relative permittivity of 79 and conductivity 0.41 S/m. In (b), the simulations in the Duke Virtual Family Adult Head Model. In (c), B_1_^+^ maps acquired in the homogeneous phantom with relative permittivity of 79 and conductivity 0.41 S/m. In (d), in-vivo human B_1_ maps. All maps are scaled to the square root of the sum of the square of all connected transmitting channels.

Fig 6 shows the simulated and measured B_1_^+^ maps for the four eigenmodes excited by each of the five Z levels of the 20-channel transmit array. Fig 6(A) and 6(C) show, respectively, the simulated and measured data in the homogeneous spherical phantom. Fig 6(B) shows the simulated B_1_^+^ maps in the Duke head model. For a visualization resembling the in-vivo acquired data, a limited number of tissues are shown: tissues distant from the brain (e.g., tongue muscle) or tissues which produce low MR signal (e.g., bone) was removed from the Fig 6(B), although the simulations were conducted using the complete Duke head model. Fig 6(D) shows the in-vivo acquired eigenmodes. The results show excellent agreement between the simulated and measured data.

### Combination of the eigenmodes

**Fig 7:**
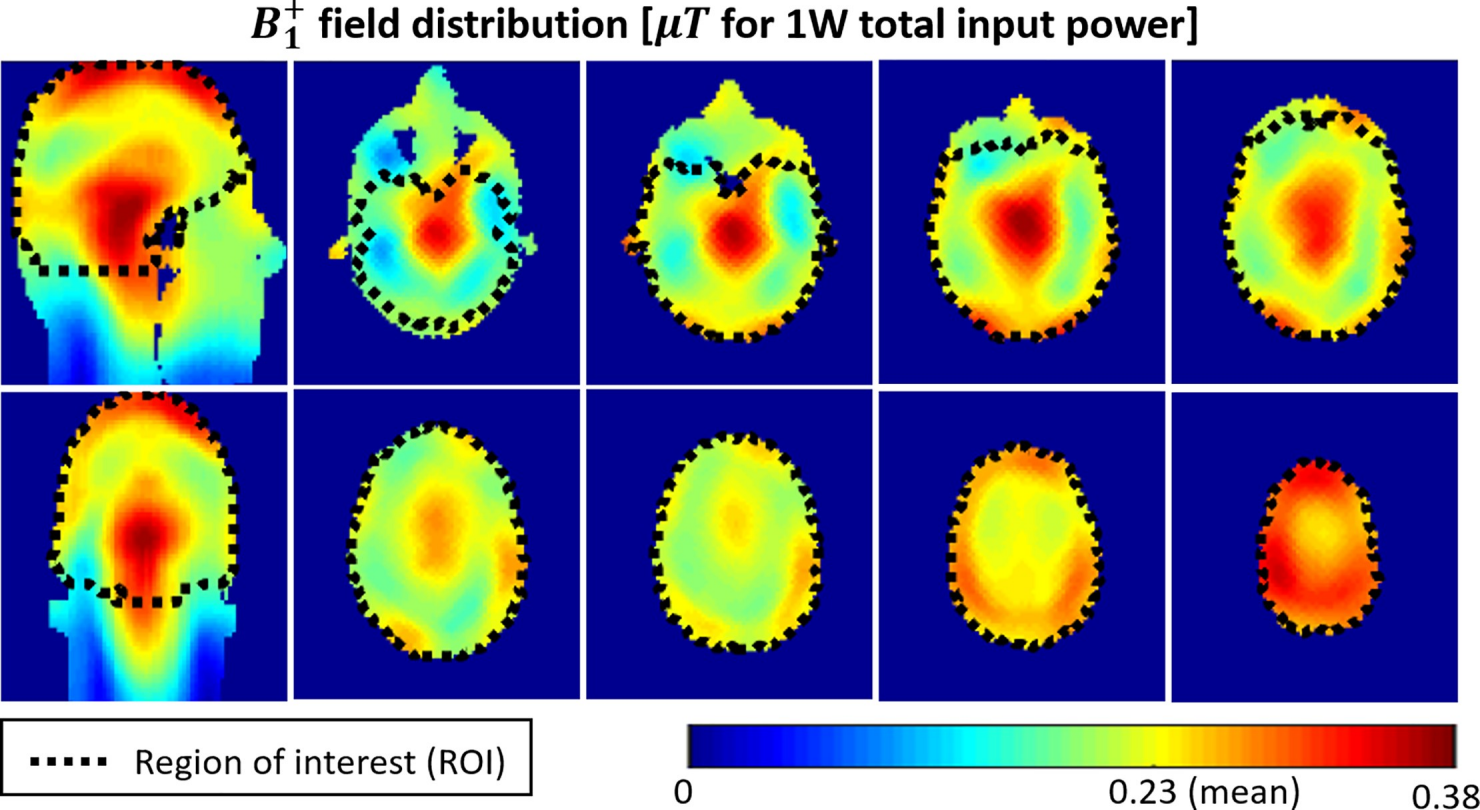
An example of the combination of the modes of the Tic-Tac-Toe coil (20 Tx channels). The ROI represents the entire head above and including the cerebellum and excluding the nasal cavities.

Fig 7 shows the combination of the modes by minimizing the CV of the B_1_^+^ fields in the ROI. The values in the ROI (composed by the whole head above and including the cerebellum and excluding the nasal cavities) are: $CV_{B_{1}^{+}}$ = 16.6%, $Max_{B_{1}^{+}}/Min_{B_{1}^{+}}$ = 3.51, SEE = 1.48 $\mu T/\sqrt{W/kg}$ (defined as mean B_1_^+^ in the ROI divided by the square root of the SAR for the whole head), mean B_1_^+^ = 0.23μT for 1W total input power.

## Discussion

In UHF MRI, as the wavelength of the electromagnetic waves inside the tissues gets closer, in size, to the body parts being scanned, inhomogeneities become a major issue, as it can affect the image quality, creating voids and low contrast regions (especially in high flip-angle sequences). In the case of brain imaging, this situation is usually accentuated in the lower brain regions such as cerebellum and temporal lobes [37]. There are several works suggesting the use of two modes to increase the homogeneity of the B_1_^+^ field distribution [13, 38, 39]. Another work suggested that the coefficient of variation of a 2D image can reach 10% by using four eigenmodes in a homogeneous spherical phantom using a birdcage RF coil [12]. At many instances, the application of these methods can come at a significant elevation of time of acquisition, elevated SAR, and difficulties in simultaneously exciting several distinct modes of a coil [34, 40, 41].

The freedom to manipulate the current distribution of different coil elements potentially contributes to the generation of a homogeneous B_1_^+^ fields distribution [42–44]. However, coil arrays typically show the capability to control current distributions only at XY plane, while current distribution are not very commonly controlled in the Z direction. It is worth noting that there are some coil designs that can potentially generate current control along the Z direction. Some examples are: the multi-rows/rings transmit arrays that allow parallel transmission approaches [45–47]; the rotating RF coil approach [48, 49]; and the spiral volume coil [50].

In this work, the eigenmodes of a 20-channel Tic-Tac-Toe RF array were studied. The RF array is composed of five excitation levels located at different positions along the static magnetic field. For each level (composed of four ports located in the same XY plane), there are four distinctive modes (with 90° phase-shift multiples) that can be generated, calculated using Eq 2. Using power splitters and phase shifters (1-to-N network), up to 5 different modes can be excited simultaneously in a single image acquisition (since each Z level can present a different excitation mode), potentially improving B_1_^+^ homogeneity and reducing SAR levels. Thus, 1024 possible combinations can be implemented using the five Z levels of the RF array, if the amplitudes of the channels are fixed.

It is important to analyze the field distribution of the eigenmodes provided by an RF head array so that a target homogeneous/low SAR excitation can be achieved. In terms of B_1_^+^ distribution inside the human head, our results show that: Mode_1 (quadrature) is the most efficient, producing center brightness at different Z levels. However, voids are observed in some regions in the lower brain (such as the cerebellum and temporal lobe regions). Mode_2 (Opposite-phase) produces low signal in the center and excites mostly the periphery regions of the brain. Mode_3 excites regions in the head periphery (mostly skin and skull) and can have localized functions such as fat suppression (extracerebral lipids from skins and skull can be suppressed to reduce the influence from this region and leave the central brain regions unaffected). Mode_4 excites mostly the lower brain regions (cerebellum and temporal lobes). The analysis also shows that Mode_1 of Level_1 and Level_4 can excite relatively uniform B_1_^+^ distributions, with CV = 22% and 22% inside ROI8 (upper head) for Level_1 and Level_4 respectively.

While there can be many solutions for the RF excitation that achieve a satisfactory signal fidelity to the targeted excitation pattern (e.g., homogeneous B_1_^+^ field), minimizing the local SAR is also an important target for the coil design and operation. In this work, the average and peak SAR was compared for different Z levels and eigenmodes. It is important to note that the SAR distribution presented in this work is an outcome of the phases and amplitudes determined by the eigenmodes, which were calculated using only the B_1_^+^ fields. Therefore, lower levels of SAR can be achieved if SAR constraints are included. Mode_1 produces lower average (< 0.11 W/kg) and peak SAR (except Top_Level), combined with efficient B_1_^+^ in the upper head, leading to a high SEE (>1.5 $\mu T\sqrt{kg}/\sqrt{W}$) as seen in the Levels 1, 2 and 4. This is higher than the double row loop array (0.76 $\mu T\sqrt{kg}/\sqrt{W}$ [36, 51]) and the birdcage coil (0.89 $\mu T\sqrt{kg}/\sqrt{W}$ [52]) for instance. While Top_level produces a high peak SAR, it is B_1_^+^ efficient, resulting in SEE of ~1.5. Mode_2 (opposite-phase) produces a relatively high SEE for Levels 2 and 3, with relatively high brain peripheral excitation. Although Mode_4 (zero-phase) presents low levels of SEE, it has efficient B_1_^+^ in the low brain regions which are challenging at UHF MRI.

The simulations were experimentally verified by acquiring the individual channels B_1_^+^ maps in the homogeneous spherical phantom and in-vivo in human subjects. The field distributions of the eigenmodes were then calculated and compared with the simulated fields (Fig 6). The modes are highly consistent between simulations and experiments. Small differences may be due to differences in the head/phantom position in simulations and experiments, differences in the tuning of the RF coil elements, differences in the hardware of the transmitting channels, and differences in the human head model and the subject scanned. Discrepancies in the B_1_^+^ maps between the phantom and the head can be mostly attributed to dielectric effects–that commonly occur in homogeneous water phantoms [53]–and to the anatomical differences between the two models.

An example of the combination (RF shimming) of the modes demonstrates a high level of the homogeneity and coverage of the B_1_^+^ field over the ROI, as demonstrated by the values of $CV_{B_{1}^{+}}$ = 16.6%, and $Max_{B_{1}^{+}}/Min_{B_{1}^{+}}$ = 3.51. The low level of SAR is also demonstrated with a high level of SEE (1.48 $\mu T/\sqrt{W/kg}$) even though SAR constraints were not included as a part of the RF shimming. The strong coupling between opposite channels (-3 to -4dB) can improve the load insensitivity of the array (being able to scan subjects with different head volumes/shapes and achieve similar RF characteristics), with the cost of lower transmit efficiency. Nevertheless, an example of the combination of the modes (Fig 7) shows that the transmit RF array produces enough B_1_^+^ intensity to perform inversion with a 1ms square RF pulse using 8kW power amplifier capability with ~35% loss to the coil port.

## Conclusions

The eigenmode arrangement of the TTT 20-channel RF array potentially allows controlling RF excitation not only at XY plane but also along the Z direction. As five eigenmodes from different Z levels can be excited simultaneously (one per excitation level in Z), we believe that the combination of these modes can provide a full brain homogeneous B_1_^+^ excitation. Future work will include the combination/superposition [6, 54–57] of these eigenmodes in order to obtain a homogeneous and efficient B_1_^+^ field distribution with low levels of SAR.
